# Factors Affecting Elective and Non-elective Cesarean Sections: A Multinomial Regression Analysis Using Robson’s Classification in a Southeast European Country

**DOI:** 10.7759/cureus.62038

**Published:** 2024-06-10

**Authors:** Arjan Shylla, Daniela Teferici, Aldo Shpuza, Xhesika Xhetani, Enver Roshi

**Affiliations:** 1 Department of Obstetrics and Gynecology, University Hospital of Obstetrics and Gynecology “Koco Gliozheni”, Tirane, ALB; 2 Department of Cardiology and Cardiac Surgery, University Hospital Center “Mother Theresa”, Tirane, ALB; 3 Department of Public Health, University of Medicine, Tirane, ALB; 4 Department of Radiology, Korca Region Hospital, Korce, ALB

**Keywords:** albania, elective, multinomial regression, robson classification, cesarean sections

## Abstract

Background: Albania, a middle-income Southeast European country, is experiencing an increase in cesarean section rates. This study aims to analyze cesarean section practices in Albania using the Robson classification to identify patterns and provide insights into elective and non-elective cesarean trends.

Methods: This retrospective cohort study was conducted at the University Hospital of Obstetrics and Gynecology “Koco Gliozheni” in Albania, a leading tertiary hospital, from January to May 2023, involving 5,315 consecutive women who delivered during this period, including both live births and stillbirths, with a gestational age minimum of 28 weeks to align with standards of viability. We defined a function to systematically evaluate each case based on multiple criteria: parity, fetal presentation, onset of labor, previous deliveries, number of fetuses, and gestational age according to the Robson classification. Multinomial multiple regression was used to estimate the relationship between each of the above-mentioned variables and the likelihood of each type of cesarean delivery compared to normal births.

Results: The participants' mean age was 28.2 years (59.6% <30 years vs. 40.4% ≥30 years), while gestational age varied (12.1% before 37 weeks, the majority (72.3%) between 37 and 40 weeks, and 15.6% > 40 weeks). In elective cesarean sections, maternal age (odds ratio (OR) = 1.06) and gestational age (OR = 1.13) were associated with increased odds, with women with previous cesarean deliveries showing significantly higher odds (OR = 20.6), breech position (OR = 15.7), and multiple pregnancies elevating odds (OR = 7.3), whereas in non-elective cesarean sections, similar associations were observed with slightly different odds ratios which were maternal age (OR = 1.07), gestational age (OR = 1.16), previous cesarean delivery (OR = 6.3), breech position (OR = 8.5), and multiple pregnancies (OR = 5.1). Significant disparities in cesarean section rates were observed across various groups, with rates ranging from as low as 0.74% in Group 1 to as high as 89.24% in Group 5, and notable contributions from Group 2 with a rate of 69.95% and Group 6 with a rate of 81.29%.

Conclusion: In conclusion, this study emphasizes the significance of factors such as maternal age, gestational age, previous cesarean deliveries, fetal presentation, number of fetuses, and multiple pregnancies in impacting the rates of elective, non-elective, and overall cesarean sections in Albania, highlighting the need for targeted strategies to improve maternal and fetal health outcomes.

## Introduction

In Albania, a Southeast European country, the escalating incidence of cesarean sections without clear medical justifications mirrors a significant global health dilemma. As a middle-income country, Albania is experiencing a trend similar to that observed in other middle-income countries such as Mexico, Turkey, Egypt, Brazil, and the Dominican Republic [1], where cesarean rates have notably increased. Similarly, this rise in cesarean sections is also evident in other Southeast European countries with socioeconomic profiles akin to Albania, including Bosnia, Macedonia, and Montenegro [2, 3]. This context underlines the significance of investigating cesarean practices in Albania, where such research is markedly lacking, despite the global inclination towards increased cesarean deliveries in countries with parallel economic backgrounds.

The epidemiological paradox in cesarean delivery is sharply illustrated by the contrasting perspectives: while the WHO recommends a 15% cesarean rate, recent studies suggest that rates of up to 19% do not increase maternal or perinatal mortality and morbidity, yet cesarean delivery carries a up to threefold greater risk of severe adverse outcomes compared to vaginal delivery, underscoring the complexity of defining an optimal approach [4,5]. Over time, the conditions for performing cesarean sections have significantly improved, as evidenced by advancements in operative techniques, anesthesia, the availability of antibiotics and blood products, and the evolving recognition of the fetus as a patient, all contributing to safer procedures and expanded indications for cesarean delivery [6, 7]. In response to the increased demand for elective cesarean sections, which partly stems from the perception that cesarean delivery is much safer now than in the past, it's important to recognize that although advancements in medical practice have made cesareans more reliable, women still choose this method for various reasons, including avoiding labor pain, fear and anxiety related to labor or birth, and the desire for a more controlled and predictable delivery process [8].

While natural childbirth remains a cost-effective and effective option for primiparous women with normal risk, cesarean sections emerge as a more efficient and equally effective choice for multiparous women with previous uterine scar [9]. Incorporating the Robson classification with its 10 group indicators is crucial for a nuanced analysis of cesarean section trends in Albania, as it not only addresses the underexplored aspects of elective and emergency cesarean sections but also aligns with WHO guidelines for standardized classification, crucial for developing targeted healthcare strategies in varying healthcare contexts [4,10,11]. Given this complex landscape of cesarean delivery in Albania, this study aims to comprehensively analyze cesarean section practices in the country, employing the Robson classification to discern patterns and decision-making criteria. This will provide crucial insights into elective and emergency cesarean trends, contributing to a better understanding of maternal healthcare needs and facilitating the development of more tailored, effective healthcare strategies in Albania's unique socioeconomic and medical context.

## Materials and methods

This study constitutes a retrospective cohort design, meticulously conducted at the obstetrics department of the University Hospital of Obstetrics and Gynecology “Koco Gliozheni,” one of the two university maternity hospitals in Tirane, Albania. The study period spanned from January 2023 to May 2023. This tertiary hospital, known for its comprehensive healthcare services, is equipped with a round-the-clock obstetrics team, pediatric services, anesthesia facilities, and a neonatal department, providing an all-encompassing healthcare environment ideal for such a study.

Study population

The study encompassed 5,315 women who delivered at the hospital during the specified period. Participants in this study were selected consecutively, ensuring a systematic inclusion of all eligible women presenting for delivery during the study period. To ensure a comprehensive analysis, the study included both live births and stillbirths, provided they met the key inclusion criterion of gestational age of at least 28 weeks. Exclusion criteria were applied to remove any cases that did not meet this gestational threshold. Furthermore, any records lacking complete data on the delivery method or those with gestational ages inaccurately recorded were also excluded.

Data collection 

Data collected from medical records and charts were meticulously coded and entered into a specially created database, facilitating detailed analysis and interpretation. Numerical variables included maternal age and gestational age, quantified respectively in years and weeks. Categorical variables included parity (nulliparous, multiparous), onset of labor (spontaneous, induced, or absent), fetal presentation (vertex, breech, transverse), previous delivery types (vaginal, cesarean, or no previous delivery), number of fetuses (singleton, multiple), and mode of delivery (elective, non-elective, or no cesarean).

Data analysis

To understand the distribution and prevalence of these variables, frequency, and percentage analyses were conducted, offering a quantitative depiction of each variable within the sample population. Furthermore, the chi-square test was employed to explore associations between categorical variables, particularly focusing on the relationship between types of delivery (elective cesarean, non-elective cesarean, or no cesarean) and other obstetric and demographic factors. Moreover, the study leveraged multinomial multiple regression analysis to delve deeper into the data, adjusting for potential confounding factors. The adjusted odds ratios (ORs) and their respective 95% confidence intervals (CIs) were calculated to estimate the relationship between each independent variable (such as maternal age, gestational age, fetal presentation, parity, and number of fetuses) and the likelihood of each type of cesarean delivery compared to normal births. The Robson classification is typically based on the criteria listed in Table 1.

**Table 1:** The Robson classification of women for cesarean sections [10]

Group	Description
1	Nulliparous, single cephalic, ≥37 weeks, in spontaneous labor
2	Nulliparous, single cephalic, ≥37 weeks, induced or cesarean before labor
3	Multiparous (excluding previous cesarean), single cephalic, ≥37 weeks, in spontaneous labor
4	Multiparous (excluding previous cesarean), single cephalic, ≥37 weeks, induced or cesarean before labor
5	Previous cesarean, single cephalic, ≥37 weeks
6	All nulliparous breeches
7	All multiparous breeches (including previous cesarean sections)
8	All multiple pregnancies (including previous cesarean sections)
9	All abnormal lie presentations (including previous cesarean section)
10	All single cephalic, <37 weeks (including previous cesarean section)

For our Robson analysis, we utilized Python's (Python Software Foundation, Wilmington, DE) powerful data processing capabilities, particularly its ability to efficiently handle conditional logic with the if-elif-else structure. This approach was pivotal in implementing Robson's Ten-Group Classification System. We defined a function to systematically evaluate each case based on multiple criteria: parity, fetal presentation, onset of labor, previous deliveries, number of fetuses, and gestational age. Each record in the dataset was passed through this function, where a series of if-elif conditions determined its classification into one of the 10 Robson groups. The entirety of this analysis was conducted using IBM SPSS Statistics Software for Windows, version 21 (IBM Corp., Armonk, NY), and Python, combining the statistical prowess and data handling capabilities of both platforms. A p-value < 0.05 was considered statistically significant.

Ethical considerations

Ethical considerations were meticulously addressed, with respect given to the confidentiality and anonymity of patient data, in line with standard research ethics and the guidelines provided by the University Hospital of Obstetrics and Gynecology “Koco Gliozheni.” All data were handled with the utmost care to maintain the privacy and dignity of the participants involved.

## Results

The descriptive statistics for maternal age in the study showed a mean age of 28.2 years (SD = 14.7) and a median age of 28 years, indicating a central tendency around the late twenties with a relatively broad age range among the participants. The maternal age distribution showed that 3,167 participants (59.6%) were under 30 years of age, while 2,148 participants (40.4%) were 30 years or older. Regarding parity, the data were nearly evenly split, with 2,642 women (49.7%) being primiparous and 2,673 women (50.3%) having two or more children. Gestational age varied, with 645 births (12.1%) occurring before 37 weeks, the majority (3,843 births, 72.3%) between 37 and 40 weeks, and 827 births (15.6%) beyond 40 weeks. The majority of births (4,999 births, 94.1%) presented in vertex position, while 277 births (5.2%) were breech, and 39 births (0.7%) were transverse. Singleton births were predominant at 5,200 births (97.8%), compared to 115 births (2.2%) for multiple births. Labor induction was spontaneous in 2,525 cases (47.5%), while 438 cases (8.2%) underwent induced labor and 2,352 cases (44.3%) had no labor. As for the mode of delivery, 1,111 cases (20.9%) were elective cesarean, 809 cases (15.2%) occurred during labor, and the majority (3,395 cases, 63.9%) did not involve a cesarean section (Table 2).

**Table 2:** Maternal demographics and birth characteristics *indicates the number (N); **indicates the percentage (%)

Variable	N^*^(%)^**^
Maternal age (years)	
<30	3167 (59.6)
≥30	2148 (40.4)
Parity	
1	2642 (49.7)
≥2	2673 (50.3)
Gestational age (weeks)	
<37	645 (12.1)
37–40	3843 (72.3)
>40	827 (15.6)
Presentation	
Vertex	4999 (94.1)
Breech	277 (5.2)
Transverse	39 (0.7)
Number of fetuses	
Singleton	5200 (97.8)
Multiple	115 (2.2)
Induction of labour	
Spontaneous	2525 (47.5)
Induced labor	438 (8.2)
No labor	2352 (44.3)
Mode of delivery	
Elective	1111 (20.9)
Non-elective	809 (15.2)
No cesarean	3395 (63.9)

Table 3, categorized by different clinical and demographic characteristics, demonstrates a significant association (p <0.001) with the type of delivery: elective cesarean section, non-elective cesarean section, or normal birth (no cesarean section). For the presentation of the fetus, vertex cases predominantly resulted in normal births (3,328, 66.6%), whereas breech and transverse presentations were more likely to require elective cesarean section (147, 53.1%, and 18, 46.2%, respectively). Parity showed a distinction where multiparas had a higher elective cesarean section rate (629, 23.5%) compared to nulliparas (482, 18.2%). The history of previous delivery was a strong indicator, especially for those with a prior cesarean, resulting in a high rate of elective cesarean section (527, 64.4%). Singleton pregnancies mostly led to normal births (3,361, 64.6%), contrasting with multiple pregnancies where elective cesarean section was more common (54, 47.0%). Maternal age affected the mode of delivery; women aged 30 years or older experienced higher elective cesarean section rates (530, 24.7%) compared to younger women. Lastly, gestational age influenced the delivery type, with the highest elective cesarean section rate observed in the 37-40 week group (941, 24.5%) and the lowest in pregnancies exceeding 40 weeks, where normal births were more prevalent (589, 71.2%) (Table 3). 

**Table 3:** Comparative analysis of delivery types across key obstetric and demographic factors ^*^indicates the number (N); ^**^indicates the percentage (%); ^†^the statistical significance is considered at p <0.05

Variable category	Elective cesarean section (N^*^, %^**^)	Non-elective cesarean section (N^*^, %^**^)	No cesarean section (N^*^, %^**^)	P-value (chi-square)^†^
Presentation				
Vertex	946 (18.9)	725 (14.5)	3328 (66.6)	P<0.001
Breech	147 (53.1)	73 (26.4)	57 (20.6)	
Transverse	18 (46.2)	11 (28.2)	10 (25.6)	
Parity				
Nullipara	482 (18.2)	525 (19.9)	1635 (61.9)	P<0.001
Multipara	629 (23.5)	284 (10.6)	1760 (65.8)	
Previous delivery				
No previous delivery	484 (18.2)	528 (19.8)	1650 (62.0)	P<0.001
Vaginal	100 (5.4)	113 (6.2)	1622 (88.4)	
Cesarean	527 (64.4)	168 (20.5)	123 (15.0)	
Number of fetuses				
Singleton	1057 (20.3)	781 (15.0)	3361 (64.6)	P<0.001
Multiple pregnancies	54 (47.0)	28 (24.3)	33 (28.7)	
Maternal age (years)				
<30	581 (18.3)	427 (13.5)	2159 (68.2)	P<0.001
≥30	530 (24.7)	382 (17.8)	1236 (57.5)	
Gestational age (weeks)				
<37	115 (17.8)	85 (13.2)	445 (69.0)	P<0.001
37–40	941 (24.5)	541 (14.1)	2361 (61.4)	
>40	55 (6.7)	183 (22.1)	589 (71.2)	

In this analysis of the predictors for elective cesarean and cesarean during labor using multinomial regression, each variable demonstrated specific influences on the likelihood of cesarean deliveries.

For elective cesarean, each additional year of maternal age raised the odds by 6.4% (OR = 1.1, 95% CI: 1.0-1.1), and each extra week of gestational age increased the odds by 13.3% (OR = 1.1, 95% CI: 1.1-1.2). The odds for nulliparous women undergoing an elective cesarean were slightly higher by 22.9% (OR = 1.2, 95% CI: 0.4-3.8). In cases of multiple pregnancies, the odds were about 7.3 times higher (OR = 7.3, 95% CI: 4.2-12.6). For fetal positions, transverse and breech increased the odds significantly, with ORs of 10.9 (95% CI: 4.2-28.5) and 15.7 (95% CI: 10.8-22.6), respectively. In the context of cesarean during labor, each year increase in maternal age had an odds increase of 7.0% (OR = 1.1, 95% CI: 1.1-1.1), and each additional week of gestation increased the odds by 16.3% (OR = 1.2, 95% CI: 1.1-1.2). For nulliparous women, the odds of cesarean section during labor increased by 35.9% (OR = 1.4, 95% CI: 0.4-4.2). Multiple pregnancies raised the odds about five-fold (OR = 5.1, 95% CI: 2.8-9.0). For fetal positions, transverse and breech also significantly increased the odds of cesarean section during labor, with ORs of 9.0 (95% CI: 3.4-24.1) and 8.5 (95% CI: 5.7-12.6), respectively (Table 4).

**Table 4:** Predictors of cesarean section types: multinomial regression analysis ^†^regression coefficient; ^‡^standard error; ^§^Wald chi-square test; ^¶^degrees of freedom; ^††^the statistical significance is considered at p <0.05; ^‡‡^exponentiated coefficient (odds ratio); ^*^reference category

Cesarean section	B^†^	Std. error^‡^	Wald^§^	df^¶^	Sig.^††^	Exp(B)^‡‡^	95% Confidence interval for Exp(B)^‡‡^
							Lower bound
Elective							
Intercept	-8.266	0.95	75.721	1	0		
Maternal age	0.062	0.008	57.762	1	0	1.064	1.047
Gestational age	0.125	0.019	45	1	0	1.133	1.093
Nullipara	0.206	0.576	0.128	1	0.721	1.229	0.397
Multipara	0b*	.	.	0	.	.	.
Previous cesarean section	3.027	0.582	27.081	1	0	20.633	6.599
Previous vaginal	-1.537	0.584	6.924	1	0.009	0.215	0.068
No previous delivery	0b*	.	.	0	.	.	.
Multiple pregnancies	1.987	0.278	51.106	1	0	7.292	4.229
Singleton	0b*	.	.	0	.	.	.
Transverse	2.392	0.489	23.936	1	0	10.937	4.195
Breech	2.75	0.188	214.005	1	0	15.65	10.826
Vertex	0b*	.	.	0	.	.	.
Non-elective							
Intercept	-9.329	0.991	88.572	1	0		
Maternal age	0.067	0.008	69.107	1	0	1.07	1.053
Gestational age	0.151	0.02	57.324	1	0	1.163	1.119
Nullipara	0.306	0.573	0.286	1	0.593	1.359	0.442
Multipara	0b*	.	.	0	.	.	.
Previous cesarean section	1.833	0.582	9.9	1	0.002	6.25	1.996
Previous vaginal delivery	-1.407	0.58	5.888	1	0.015	0.245	0.079
No previous delivery	0b*	.	.	0	.	.	.

In the comprehensive analysis of cesarean section rates across different groups, a notable distinction emerges between elective and non-elective cesarean sections during labor. Group 1, with no elective cesarean sections and only eight during labor cases, had the lowest cesarean rate of 0.74%, contributing minimally (0.42%) to the overall rate despite comprising 20.4% of the sample. Conversely, Group 5 exhibited the highest rate of cesarean sections at 89.24%, with 453 elective and 144 during labor cases, significantly contributing 31.09% to the overall rate while representing 12.6% of the sample. Group 2 also displayed a high cesarean section rate of 69.95%, with 321 elective and 424 during-labor cases being a major contributor (38.80%) to the overall rate. Similarly, Group 3 had no elective cesarean sections but 10 during-labor cases, resulting in a low rate of 0.81% and a minor overall contribution of 0.52%. Additionally, other groups exhibit varied cesarean section patterns. For instance, Group 4, with a moderate overall cesarean rate of 36.46%, comprised 60 elective and 76 during-labor cases, contributing 7.08% to the total rate. Group 6, having 82 elective and 44 during labor cases, presented a high cesarean rate of 81.29%, yet constituted only a small fraction (2.9%) of the sample, with a contribution of 6.56% to the overall rate. Groups 7, 8, and 10 also showed distinct cesarean patterns, with Group 7 having a high rate of 75.28% (46 elective, 21 during labor) and Groups 8 and 10 displaying rates of 70.69% and 24.95%, respectively, with Group 8 contributing 4.27% and Group 10 contributing 6.56% to the overall cesarean rate (Table 5). 

**Table 5:** Cesarean section analysis according to Robson classification groups CS: cesarean section ^*^The first four columns (elective CS, during-labor CS, no CS, and total deliveries) are numbers; ^†^the last three columns (rate of CS in each group, relative size in each group, contribution of each group to overall CS rate) are percentages.

Group	Elective CS^*^	During-labor CS^*^	No CS^*^	Total Deliveries^*^	Rate of CS in each group (%)^†^	Relative size in each group (%)^†^	Contribution of each group to overall CS rate (%)^†^
1	0	8	1075	1083	0.74	20.4	0.42
2	321	424	320	1065	69.95	20	38.8
3	0	10	1217	1227	0.81	23.1	0.52
4	60	76	237	373	36.46	7	7.08
5	453	144	72	669	89.24	12.6	31.09
6	82	44	29	155	81.29	2.9	6.56
7	46	21	22	89	75.28	1.7	3.49
8	54	28	34	116	70.69	2.2	4.27
9	16	7	10	33	69.7	0.6	1.2
10	79	47	379	505	24.95	9.5	6.56

## Discussion

This study, conducted in the unique context of Albania's healthcare system, reveals significant trends and variations in cesarean section practices, underscoring the necessity for tailored approaches in maternal healthcare, informed by the insights derived from the Robson classification and multinomial regression analysis.

Key findings and comparisons with international studies

Albania, from 2018 to 2023, has had an average of about 27,500 births per year, while our study encompassed a cohort of 5,315 patients at the "Koco Gliozheni" Hospital, which accounts for 15%-20% of all births in the country [12]. Our study shows a mean maternal age of 28.24 years, with a majority (59.6%) of participants under 30 years, suggesting a younger maternal population. In contrast, a study in France reports a higher mean maternal age of 31.9 years, with a greater proportion (55.9% vs. 49.7%) of nulliparous women, differences that could reflect regional or demographic differences in reproductive patterns [13]. In comparing our Albanian data with a comprehensive study from Brazil, both being middle-income economies, we observe that Brazil's cesarean section rates exhibit a distinct age-related trend, with rates escalating from 40% in women under the age of 20 to a notable 69.4% in those aged 35-39, reflecting a tendency towards increased surgical interventions as maternal age rises [14]. Additionally, the similarity in the predominance of singleton births is evident in both countries, with Brazil reporting 97.8% of singleton births, paralleling our findings. However, variations are notable in gestational age distributions and labor induction practices, where our study indicated 72.3% of births occurring between 37 and 40 weeks and spontaneous labor initiation in 47.5% of cases, differing slightly from the Brazilian context, thus highlighting the diverse obstetric landscapes shaped by regional healthcare practices and demographic characteristics [14,15]

In our study, the marked tendency for elective cesarean sections in cases of breech and transverse fetal presentations, as well as the higher cesarean rates among multiparous women compared to nulliparas, resonates with findings from another study, which highlights non-cephalic presentation and the absence of a history of vaginal delivery as significant predictors for opting for cesarean delivery [16]. Comparing our study with another recent hospital-based, cross-sectional study, we find interesting parallels and distinctions in the factors influencing cesarean section decisions. We observed a higher rate of elective cesarean sections for breech (53.1%) and transverse (46.2%) fetal presentations, which aligns with the findings of the other study; however, our study contrasts with theirs in terms of primiparous women; while they found a higher incidence of emergency cesarean sections in primigravida women (p <0.001), our data indicated a marginally increased likelihood of elective cesarean sections in this group (18.2%) [17].

Factors influencing cesarean section decisions

The multinomial regression analysis in our study offers a detailed exploration of factors influencing cesarean section outcomes, with natural birth serving as the reference category. This sophisticated statistical approach enables a comprehensive understanding of how each variable differentially affects the odds of undergoing an elective versus non-elective cesarean section.

For instance, maternal age increases the odds of both elective (OR = 1.064) and non-elective cesarean section (OR = 1.070) compared to natural birth, suggesting a consistent age-related risk across different cesarean section types. However, the slightly higher OR in the non-elective category underscores the increased urgency or complication risk as age advances, particularly in emergent situations. Reflecting on a similar study from a tertiary hospital in Switzerland, it was found that nulliparous pregnant women over the age of 40 had a higher risk of unscheduled cesarean delivery, with an adjusted odds ratio of 1.53 (95% confidence interval, 1.06-2.19) compared to younger women [18].

Gestational age showcases a more pronounced difference, with a 13.3% and 16.3% increase in the odds for elective (OR = 1.13) and during-labor cesarean sections (OR = 1.16), respectively. This indicates gestation’s critical role, with overdue pregnancies leaning more towards during-labor interventions, possibly due to complications arising late in the term that necessitate immediate action. Decision-making in maternal healthcare, which affects cesarean section rates, is also influenced by sociodemographic profiles as well as cervical-vaginal infections in mothers at risk of preterm birth, as highlighted by another study in Albania [19]. The Exp(B) value for a previous cesarean significantly elevates the odds of an elective cesarean section (OR = 20.63), underscoring the principle of "once a cesarean, often a cesarean," which is reflective of both medical guidance and patient choice [20]. Conversely, a history of vaginal delivery drastically reduces the odds of an elective cesarean section (OR = 0.21), highlighting the confidence in vaginal birth after cesarean (VBAC) as a viable and safe option for many women.

The distinction between multiple and singleton pregnancies is another critical determinant. Multiple pregnancies are associated with a significantly higher likelihood of elective cesarean delivery (OR = 7.29), which is consistent with the medical literature emphasizing the increased risks and complexities involved in delivering multiples. The lower odds ratio for emergency cesarean sections in our study, compared to elective ones, mirrors the nuanced approach to managing multiple gestations, where elective cesarean is often preferred to address the heightened risk of maternal postpartum complications associated with vaginal delivery of twins or higher-order multiples, complications that not only stem from the surgery itself but also from preexisting conditions and earlier delivery times [21].

Robson classification analysis and its implications for cesarean section practices

A systematic review of the use of the Robson classification for cesarean section highlighted its rapid and spontaneous adoption worldwide, citing its simplicity, robustness, and flexibility as major strengths [22]. In our study, the classification according to the Robson system allowed for a detailed examination of cesarean section rates across various groups. Group 1, predominantly low-risk first-time mothers, contributed minimally to the overall cesarean rate, highlighting the low cesarean necessity in this cohort. Conversely, Group 5, characterized by multiparous women with previous cesareans, exhibited the highest rate, underscoring the significant impact of obstetric history on current delivery methods. Group 2, involving nulliparous women with induction or pre-labor cesareans, also showed a high cesarean rate, indicating the influence of labor management practices. Consistent with our observations, a study conducted in Addis Ababa, Ethiopia, highlighted groups involving nulliparous women with induction or pre-labor cesareans (Group 2) and multiparous women with previous cesareans (Group 5) as significant contributors to the cesarean section rates [23].

Moreover, another study from a private tertiary hospital in Nigeria reported that Robson Group 5 had the largest contribution to the total cesarean section rates, emphasizing the impact of previous cesarean deliveries on the decision for subsequent cesarean sections [24]. The cesarean section rates across Groups 3 and 4 illustrate how labor management and obstetric history influence delivery decisions, with spontaneous labor in multiparous women leading to significantly lower cesarean rates compared to those with induced labor or previous cesareans [25]. High cesarean rates in Groups 6 and 7 for breech presentations and in Groups 8, 9, and 10 for multiple pregnancies, abnormal lies, and preterm deliveries underscore a preference for cesarean delivery in managing complex or high-risk obstetric scenarios [26].

This discussion underscores the necessity for tailored approaches in maternal healthcare, informed by robust analytical models like the Robson classification. The insights derived from these analytical techniques should guide policymakers and healthcare providers in Albania and similar contexts to optimize cesarean practices and improve maternal and neonatal outcomes.

Strengths and limitations

The study's strong point is its innovative use of the Robson classification in Albania and multinomial regression methods, revealing striking contrasts in elective and non-elective cesarean rates across risk groups, and guiding targeted healthcare interventions. The study's main limitations include its retrospective nature, focusing solely on data from a single tertiary hospital in Albania, which may limit the generalizability of findings. Additionally, potential biases in the accuracy and completeness of medical records and the inherent limitations of retrospective designs may affect the robustness of conclusions. While effective, the study’s reliance on the Robson classification and multinomial regression analysis might not capture the full nuances of individual cases.

## Conclusions

In conclusion, this study delineates the distinct influences of maternal age, gestational age, previous cesarean deliveries, fetal presentation, and multiple pregnancies on elective and non-elective cesarean sections within the Albanian context. It showcases the differential impact of these factors, with previous cesarean deliveries and non-cephalic fetal presentations significantly elevating the odds for both types of cesarean sections, albeit with varying magnitudes. The analysis, refined through the lens of the Robson classification, highlights how certain groups, particularly women with a history of cesarean delivery and those presenting with multiple or malpositioned fetuses, face a higher propensity for cesarean delivery, whether elective or emergent. Conversely, low-risk groups, such as nulliparous women with cephalic, term pregnancies initiating labor spontaneously, exhibit the lowest cesarean rates. This delineation of risk factors underscores the imperative for nuanced, risk-adjusted approaches to cesarean section management. By identifying specific areas of high cesarean incidence and understanding the variable influences on elective versus non-elective cesarean decisions, healthcare providers can tailor interventions more effectively, aiming to optimize maternal and fetal outcomes while adhering to international cesarean section guidelines.
